# Implementing GPT‐4 Learning Models in Dermatology: An Assessment of Medical Quality and Utility

**DOI:** 10.1111/srt.70331

**Published:** 2026-02-18

**Authors:** Aryan Naik, Peter Vien, Thanh‐Nga Tran

**Affiliations:** ^1^ Larner College of Medicine, The University of Vermont Burlington Vermont USA; ^2^ Wellman Center for Photomedicine, Harvard Medical School Massachusetts General Hospital Boston Massachusetts USA

**Keywords:** AI, AI in dermatology, artificial intelligence, ChatGPT

## Abstract

**Background:**

Artificial intelligence, including large language models (LLMs) such as GPT‐4, can generate responses to clinical queries using predictive algorithms trained on large online datasets. Current literature lacks a comprehensive assessment of the medical quality and accuracy of dermatologic GPT‐4‐generated outputs.

**Methods:**

A standardized query was used to ask GPT‐4 models (Copilot and ChatGPT‐4) to generate summaries and treatment recommendations for 33 dermatologic conditions, which were then compared to corresponding sections of UpToDate (UTD) excerpts. DISCERN scores were calculated for each source by two authors (AN and PV). Concordance between GPT‐4‐generated treatments and UTD was evaluated by a certified dermatologist. Word count and Flesch Kincaid reading score were generated in R. Paired *t*‐tests and one‐way and weighted ANOVA were conducted in R.

**Results:**

The DISCERN instrument classified UTD content as being of “fair” medical quality (mean [SD], 3.08 [0.34]), while both ChatGPT‐4 and Copilot produced content of “poor” medical quality (mean [SD], 2.28 [0.22] and mean [SD], 2.31 [0.35], respectively). ChatGPT‐4's treatment recommendations demonstrated 33.5% greater average concordance with UTD treatment recommendations (mean [SD], 64.89% [29.29]), in comparison to Copilot (mean [SD], 31.38% [31.08%]); (95% CI, 22.3%–44.7%, *p* < 0.001).

**Conclusions:**

Overall, GPT‐4 models produced dermatological content with few harmful recommendations. However, GPT‐4‐generated content performed poorly on the DISCERN instrument, and validation of LLM‐generated responses remains challenging. Results suggest LLM parameters and query structures may be optimizable for dermatologic applications. If implemented alongside the professional judgement of certified dermatologists, future LLMs may serve as time‐saving dermatologic tools, enhancing patient care.

**Abbreviations**
AI: artificial intelligence
LLM: large language model
UTD: UpToDate

## 1 Introduction

The emergence of large language models (LLMs) such as ChatGPT and Copilot can radically transform the landscape of healthcare. These artificial intelligence (AI) models are trained on large online datasets, and when queried, can generate clinical answers. They can also be utilized to reduce the current healthcare administrative load, screen for cutaneous cancers, diagnose lesions, and develop treatment plans for teledermatology practitioners [1, 2, 3]. Given the notable disparity in access to dermatologic expertise within remote and underserved areas, generative AI may be able to assist first‐contact providers where dermatologic resources are scarce [4, 5]. Generative AI models can help bridge the gap in dermatologic care in primary care and non‐specialist settings by facilitating treatment planning and improving diagnostic accuracy [6].

Previous iterations of ChatGPT have been effective in synthesizing differential diagnoses with an accuracy rate of 88% when presented with clinical vignettes [7]. These models have achieved scores over 50% across all US STEP examinations, with some metrics exceeding 60% accuracy, all without specialized medical training [8]. Within dermatology, ChatGPT‐4 accurately answered 90.5% of the questions on the United Kingdom Dermatology Specialty Certificate Examination [9]. ChatGPT models can even outperform providers in certain dimensions, scoring 9.8 times higher in both quality and empathy in written responses compared to physicians [10]. Despite these promising advancements for the use of AI in healthcare, it is essential to establish a robust regulatory framework to safeguard patient health and safety. Such oversight is necessary to mitigate risks arising from inconsistent performance, inherent bias, poor‐quality training data, and obscure algorithmic decision‐making [11].

While ChatGPT‐4 has demonstrated utility in dermatologic treatment planning and diagnosis, the accuracy and caliber of its outputs as physician resources for dermatologic treatment planning have not yet been assessed. This study investigates the quality of dermatologic AI‐generated healthcare treatment plans using the DISCERN scoring system and utilizes UpToDate (UTD) as a benchmark for treatment plan accuracy.

## 2 Methods

### 2.1 GPT‐4 Query

Thirty three frequently encountered dermatologic conditions were selected based on previous studies documenting the conditions most commonly seen by dermatologists, non‐dermatologists, and in teledermatology settings [12, 13]. The standardized prompt used to query ChatGPT‐4 and Copilot GPT‐4 was developed to emulate a clinical treatment question and to elicit a response that facilitates a direct comparison to the “Introduction” and “Summary and Recommendations” sections of corresponding UTD articles [14]. These sections are prominently linked at the top of each article for ease of access and synthesize key treatment information for clinicians. The prompt created was as follows: “Please provide me with a summary of the dermatologic condition ‘___.’ For this condition, provide me with a list of treatment recommendations and special considerations for managing this condition based on the severity of presentation.”

### 2.2 Statistical Analysis

DISCERN is a 16‐question standardized instrument used to assess the quality of health literature, evaluating aspects including the reliability of the publication, the quality and quantity of treatments, and the sources used [15]. Under the expertise of Dr. Thanh‐Nga Tran, authors A.N. and P.V. utilized The DISCERN Handbook to produce scores for each of the GPT‐4 outputs and UTD excerpts [16]. DISCERN scores range from 4.2 to 5.0 (excellent), 3.4 to 4.2 (good), 2.5 to 3.4 (fair), and < 2.5 being poor. Interrater reliability was calculated with Python. A kappa score of 0.41–0.60 indicates moderate agreement, while a score of 0.61–0.80 is considered substantial agreement [17].

UTD treatment guidelines served as the reference standard for evaluating the accuracy of LLM‐generated treatment recommendations. A board‐certified dermatologist assessed concordance by matching LLM treatment suggestions to UTD guidelines. In order to gauge the clinical impact and relevance of unmatched LLM treatment suggestions, they were reviewed for evidence of clinical efficacy in the literature, as well as their mechanism and safety. They were then classified as either benign (providing clinical benefit, or at minimum, unlikely to adversely impact the patient's health) or harmful (may adversely impact the patient's condition or safety). Flesch–Kincaid (FK) analysis of LLM and UTD text was performed in R. Scores were used to evaluate the literature complexity and target audience. Paired *t*‐test, one‐way and weighted ANOVA, and Tukey's HSD were performed in R.

## 3 Results

### 3.1 DISCERN Scores

Interrater reliability revealed moderate to substantial agreement. Copilot's average DISCERN score (mean [SD], 2.31 [0.35]) was lower than that of UTD's (mean = 3.08, SD = 0.34; mean difference = 0.56, 95% CI, 0.37–0.74, *p* < 0.001). Additionally, ChatGPT‐4's average DISCERN (mean [SD], 2.28 [0.22]) scored lower than UTD (mean = 0.58, 95% CI, 0.39–0.77, *p* < 0.001). No statistically significant difference was identified between the DISCERN scores for ChatGPT and Copilot.

### 3.2 Readability Scores

Average word count varied significantly between all groups: ChatGPT‐4 (mean [SD], 350.06, [39.96]), Copilot (mean [SD], 119.82, [36.86]), and UTD (mean [SD] 551.6, [189.94]); (*F* = 117.9, *p* < 0.001). ChatGPT‐4 produced content at a lower average FK reading score (mean [SD], 18.54964, [5.86]) compared to UTD (mean [SD], 15.39 [1.63]) and Copilot (mean [SD], 13.26, [36.86]). Although significant differences exist between at least two groups (*F* = 15.64, *p <* 0.001), all three scores fall within the range of the “college graduate” level of complexity (10–30).

### 3.3 Concordance to UTD

UTD produced a higher average number of suggested treatments (mean [SD], 6.24 [3.61]) in comparison to Copilot (mean [SD], 4.18 [2.66]) and a lower number in comparison to ChatGPT (mean [SD], 9.15 [3.35]). However, only ChatGPT‐4 suggested a significantly higher number of treatments in comparison to Copilot (mean = 3.12, 95% CI, 1.23–5.02, *p* < 0.001). ChatGPT‐4 responses did not include sources. Copilot provided an average of 4.18 sources (SD, 2.66) per response, 3.52 more sources in comparison to UTD (mean [SD], 1.4 [1.92]), (95% CI, 2.77–4.26, *p* < 0.001). ChatGPT‐4's treatment recommendations displayed an average 33.5% higher concordance with UTD guidelines (mean [SD], 64.89% [29.29%]) versus Copilot (mean [SD], 31.38% [31.08%]); (95% CI, 22.34%–44.67%, *p* < 0.001). Of the treatments recommended by ChatGPT‐4 and Copilot GPT‐4 but not mentioned within UTD, 99.39% and 98.53%, respectively, were benign. The proportion of harmful discordant treatments recommended by ChatGPT‐4 and Copilot outputs was 0.6% and 1.47%, respectively. Table 1 summarizes the results.

**TABLE 1:** Characteristics of responses to dermatologic queries using GPT‐4 learning models in comparison to UpToDate articles.

Characteristic	OpenAI ChatGPT‐4	Copilot GPT‐4	UpToDate	*F*	*Pr* (*>F*)
Most recent literature update	September 2021	September 2021^a^	August 2023		
DISCERN score, mean (SD)	2.28 (0.22)	2.31 (0.35)	3.08 (0.34)	23.75	**< 0.001** ^d^
Word count, mean (SD)	350.06 (39.96)	119.82 (36.86)	551.56 (189.94)	117.9	**< 0.001** ^e^
Flesch–Kincaid Reading Score, mean (SD)	18.55 (5.86)	13.26 (2.84)	15.39 (1.63)	15.65	**< 0.001** ^f^
Sources^b^ provided, mean (SD)	0 (0)	4.82 (1.04)	1.5 (1.92)	538	**< 0.001** ^g^
Proposed treatments^c^, mean (SD)	9.15 (3.35)	4.18 (2.66)	6.24 (3.61)	7.77	**< 0.001** ^h^
**Concordance to UTD**				***t***	***p* value**
GPT‐4 treatment concordance with UpToDate, mean % (SD)	64.89 (29.29)	31.38 (31.08)		6.14	**< 0.001** ^I^
No. unmatched treatments, benign (%)	165 (99.39)	67 (98.53)			
No. unmatched treatments, harmful (%)	1 (0.6)	1 (1.47)			

^a^ CopilotGPT4 version tested can access the internet through Microsoft's search engine.

^b^ *Sources* include links and citations.

^c^ Proposed treatments include any therapy which is curative, may provide symptomatic relief, or reduce disease severity.

^d^ 5.21E‐09

^e^ 2 E‐16

^f^ 0.0000013

^g^ 2 E‐16

^h^ 0.000748

^i^ 1.0969E‐06

## 4 Discussion

The powerful analytical capabilities of AI‐driven machine learning are transforming dermatology. An application developed by Google Health diagnosed images of 26 conditions with similar accuracy to that of board‐certified dermatologists [12]. This may allow for more accurate diagnosing in primary care settings and enhance patient triage. ChatGPT can also create dermatologic patient materials, reduce clinical administrative burden, and pass licensing exams [9, 18]. However, its reliability as a dermatologic resource to physicians, such as UTD, is unknown. Our study demonstrates that GPT‐4 LLMs can quickly produce large quantities of accurate and safe dermatologic treatment information. With further medical training and implementation of healthcare AI guidelines, LLMs can be leveraged by physicians and providers by creating treatment plans, streamlining literature review, and distilling the latest research for clinical practice.

Responses generated by ChatGPT‐4 and Copilot GPT‐4 exceeded UTD in certain metrics (ChatGPT‐4 provided a significantly higher number of treatments than UTD, and Copilot GPT‐4 provided a significantly greater number of sources than UTD); however, they both yielded significantly lower DISCERN scores. The lower DISCERN scores of LLM‐generated text indicate overall poorer health literature quality and raise concern for the reliability and quality of AI‐generated health information. This includes, but is not limited to, the lack of lines of therapy, discussion of treatment risks and benefits, as well as strong source citation and transparency. Despite being of inferior medical quality, LLM‐generated responses and UTD scored within the same Flesch‐Kincaid readability index. These findings confirm LLM content was of equivalent textual complexity to established reference materials and would demand a comparable degree of clinical expertise for interpretation. This further underscores that AI‐generated resources cannot be used in isolation; their limited reliability and complexity necessitate expert contextualization to achieve clinical utility.

In addition, our analysis reveals that even between GPT‐4 models, treatment adherence to UTD guidelines may vary. While both LLMs scored poorly on DISCERN, ChatGPT‐4's higher concordance rate to UTD recommendations suggests that it may be better suited for clinical reasoning and highlights the impact of fine‐tuning, even when the underlying infrastructure may be identical. However, this does not inherently mean that unmatched treatments are not effective. UTD articles do not contain an exhaustive list of therapies, and the majority of “unmatched” treatments proposed by the tested LLMs were beneficial, or at least harmless. For example, ChatGPT included imiquimod as a treatment for basal cell carcinoma (BCC), an effective treatment option not stated within UTD's article on BCC treatment.

Unlike standalone ChatGPT‐4, Microsoft's Prometheus technology allows Copilot to rapidly integrate with Microsoft's search engine, Bing, and generate source citations. Microsoft claims this technique, called “Grounding,” creates more accurate responses by synthesizing outputs with URL citations [19]. Theoretically, the ability to access the latest literature would grant Copilot significant advantages in providing the most current evidence‐based treatment options, yet this did not result in an improved average concordance rate. This may be attributed to Copilot's integration with Bing's proprietary ranking and indexing of web content, which adds another layer of bias and complexity when verifying medically reliable information. While the results indicate GPT‐4 LLMs are not yet suitable for standard clinical practice, it is essential to note that these models were created for generalized use and that refinement of the training process or prompts may improve medical reasoning.

Medicine has been justifiably wary in accepting AI models into clinical practice. LLMs are prone to “hallucinate,” creating misleading information with unwarranted confidence. LLMs often struggle with contextual understanding, a crucial aspect in providing effective patient care [20]. LLM methodology interprets characters as probabilistic functions, making it challenging to differentiate between erroneous and factual inputs. While they excel in knowledge‐based testing and complex calculation, the limited insight we have regarding their input transformation obstructs attempts to validate and reproduce their responses [21]. The inclusion of sources by Copilot simplifies the process of verifying outputs, yet further research is needed to determine its efficacy within the use‐case of medicine.

Current issues in algorithmic bias and generalizability cannot be overlooked. For instance, ProPublica's analysis revealed that COMPAS, a tool used to calculate criminal recidivism, demonstrated a bias towards African Americans [22]. Similarly, Google's photo recognition tool misidentified African Americans as gorillas [23]. This underscores the profound and potentially harmful consequences of bias in algorithms. Additionally, a recent study evaluating GPT‐4's ability to diagnose skin conditions found that its accuracy was significantly lower for darker skin tones compared to lighter ones [24]. Additionally, both GPT‐4 LLMs tested neglected to differentiate treatments or provide risks for pathologies in skin of color. This highlights the critical need to train LLMs on more diverse datasets representative of all Fitzpatrick skin types. Achieving inclusive training data demands a coordinated effort to develop publicly accessible datasets. This will drive the equitable training and performance of LLMs on darker skin tones, which has been demonstrated with fine‐tuning on diverse data [25]. Without inclusive training data, we cannot ensure that AI systems will perform equitably across all skin tones. These generalizations and inaccuracies posed by current LLMs may reflect societal prejudices and thus necessitate careful research stewardship to prevent the perpetuation of these biases.

In order to integrate LLMs into clinical medicine, it is paramount that we ensure the consistency and reliability of the information generated. This includes the need for tailored data training but also systematic frameworks for implementation that prioritize rigorous AI governance, including frequent external performance evaluation and IT support [26, 27]. Continuous monitoring and validation will ultimately help improve transparency, reduce bias, and ultimately build trust in AI systems [28]. When querying LLMs in a clinical context, we recommend using a standardized query format that explicitly requests details on treatment risks, benefits, and verifiable source citations. Although instances of harmful dermatologic recommendations were rare in our study, it remains crucial to incorporate fail‐safes within the decision‐making process. As LLMs are trained on vast datasets, AI‐generated recommendations should undergo external validation with evidence‐based guidelines specific to the clinician's country of practice. Additionally, engineering prompts, which require the AI to provide its rationale, may provide insight into the strength of evidence for each recommendation. Lastly, it is imperative that clinicians understand the limitations to AI‐generated dermatologic suggestions and know when to seek input from specialists within the field.

## 5 Conclusion

Future LLMs with optimized clinical training may serve as trustworthy resources for dermatologic information. Generative AI's ability to rapidly analyze and synthesize vast amounts of material may enhance the efficiency of literature reviews and improve physicians’ ability to incorporate evolving research into their practice. In turn, this would allow physicians to allocate greater time to patients or tasks of higher complexity [29]. It is crucial to underscore that AI models should not supplant clinical evaluations by certified dermatologists but rather offer support as one of many clinical tools. In scenarios where there exists a paucity of specialized dermatologic care, such as rural settings, emergency departments, or primary care settings, AI can provide safe and valuable treatment options or assist in visual diagnosis. Regardless, concerns regarding algorithmic bias, misinformation, and HIPAA compliance pose threats to patient safety and necessitate full ethical guidelines before these models can be considered safe for clinical practice. While the integration of generative AI into clinical practice should be approached cautiously, these results indicate promise for future implementation.

## Ethics Statement

No ethical approvals or informed consent procedures were required for this study, as it did not involve direct experimentation with human subjects or the use of sensitive personal data.

## Conflicts of Interest

The authors declare no conflicts of interest.

## Data Availability

The data that support the findings of this study are available from the corresponding author upon reasonable request.
